# Ethyl 6-methyl-2-sulfanyl­idene-4-[4-(trifluoro­meth­yl)phen­yl]-1,2,3,4-tetra­hydro­pyrimidine-5-carboxyl­ate

**DOI:** 10.1107/S1600536811019441

**Published:** 2011-06-04

**Authors:** Susanta K. Nayak, K. N. Venugopala, Thavendran Govender, Hendrik G. Kruger, Glenn E. M. Maguire, T. N. Guru Row

**Affiliations:** a Solid State and Structural Chemistry Unit, Indian Institute of Science, Bangalore 560 012, India; b School of Chemistry, University of KwaZulu-Natal, Durban 4000, South Africa; c School of Pharmacy and Pharmacology, University of Kwazulu-Natal, Durban 4000, South Africa

## Abstract

The title compound, C_15_H_15_F_3_N_2_O_2_S, adopts a conformation with an intra­molecular C—H⋯π inter­action. The dihedral angles between the planes of the 4-(trifluoro­meth­yl)phenyl and ester groups with the plane of the six-membered tetra­hydro­pyrimidine ring are 81.8 (1) and 16.0 (1)°, respectively. In the crystal structure, inter­molecular N—H⋯S hydrogen bonds link pairs of mol­ecules into dimers and N—H⋯O inter­actions generate hydrogen-bonded mol­ecular chains along the crystallographic *a* axis.

## Related literature

For applications of multi-functionalized dihydro­pyrimidines, see: Jauk *et al.* (2000 ▶); Kappe (2000 ▶); Mayer *et al.* (1999 ▶). For structural analysis, see: Nayak *et al.* (2009 ▶, 2010 ▶).
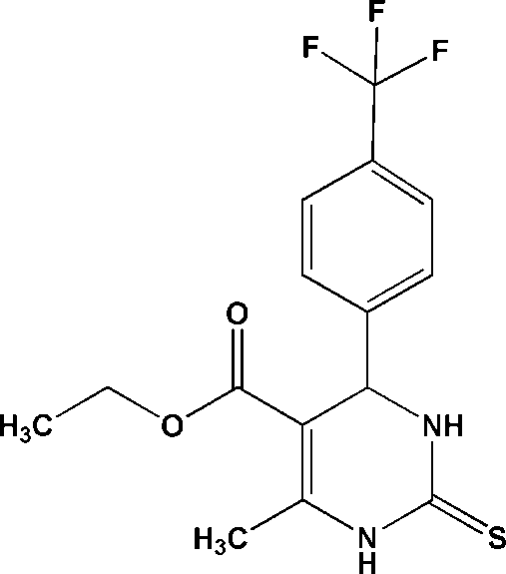


## Experimental

### 

#### Crystal data

C_15_H_15_F_3_N_2_O_2_S
*M* *_r_* = 344.36
Triclinic, 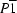

*a* = 7.2682 (3) Å
*b* = 9.3205 (2) Å
*c* = 12.4779 (3) Å
α = 74.199 (2)°
β = 88.092 (3)°
γ = 69.377 (3)°
*V* = 759.39 (5) Å^3^
*Z* = 2
Mo *K*α radiation
μ = 0.26 mm^−1^
*T* = 120 K
0.28 × 0.22 × 0.17 mm

#### Data collection

Oxford Diffraction Xcalibur diffractometer with an Eos (Nova) detector
Absorption correction: multi-scan (*CrysAlis PRO*; Oxford Diffraction, 2009 ▶) *T*_min_ = 0.932, *T*_max_ = 0.958
16986 measured reflections
2982 independent reflections
2670 reflections with *I* > 2σ(*I*)
*R*_int_ = 0.034

#### Refinement

*R*[*F* ^2^ > 2σ(*F* ^2^)] = 0.032
*wR*(*F* ^2^) = 0.085
*S* = 1.05
2982 reflections
268 parameters
All H-atom parameters refined
Δρ_max_ = 0.33 e Å^−3^
Δρ_min_ = −0.26 e Å^−3^

### 

Data collection: *CrysAlis PRO* (Oxford Diffraction, 2009 ▶); cell refinement: *CrysAlis PRO*; data reduction: *CrysAlis PRO*; program(s) used to solve structure: *SHELXS97* (Sheldrick, 2008 ▶); program(s) used to refine structure: *SHELXL97* (Sheldrick, 2008 ▶); molecular graphics: *ORTEP-3 for Windows* (Farrugia, 1997 ▶) and *CAMERON* (Watkin *et al.*, 1993 ▶); software used to prepare material for publication: *PLATON* (Spek, 2009 ▶) and *PARST* (Nardelli, 1995 ▶).

## Supplementary Material

Crystal structure: contains datablock(s) global, I. DOI: 10.1107/S1600536811019441/si2356sup1.cif

Structure factors: contains datablock(s) I. DOI: 10.1107/S1600536811019441/si2356Isup2.hkl

Supplementary material file. DOI: 10.1107/S1600536811019441/si2356Isup3.cml

Additional supplementary materials:  crystallographic information; 3D view; checkCIF report

## Figures and Tables

**Table 1:** Hydrogen-bond geometry (Å, °)

*D*—H⋯*A*	*D*—H	H⋯*A*	*D*⋯*A*	*D*—H⋯*A*
N1—H1*N*⋯O1^i^	0.80 (2)	2.20 (2)	2.9868 (19)	169.8 (18)
N2—H2*N*⋯S1^ii^	0.830 (19)	2.462 (19)	3.2830 (14)	170 (2)
C14—H14⋯*Cg*1	0.94 (2)	2.655 (2)	3.129 (2)	112

Symmetry codes: (i) 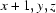; (ii) 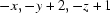.

**Fig. 1.**
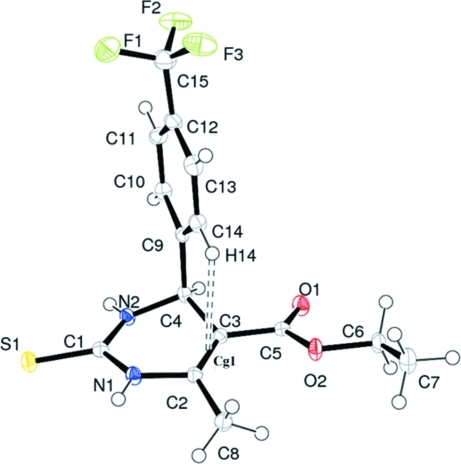
Molecular structure shows the atom labelling scheme with displacement ellipsoids for non-H atoms at 50% probability level, hydrogen atoms are arbitary circle. The dotted line shows the C—H···π intramolecular interactions. Cg1 denotes the center of gravity of the C2=C3 bond.

**Fig. 2.**
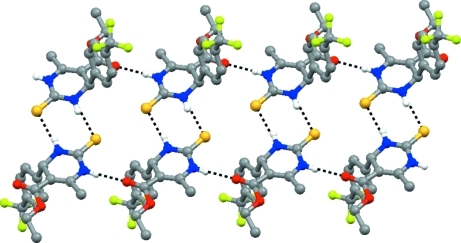
The molecular packing shows the molecular chain of N—H···O hydrogen bonds and N—H···S intermolecular dimers.

**Fig. 3.**
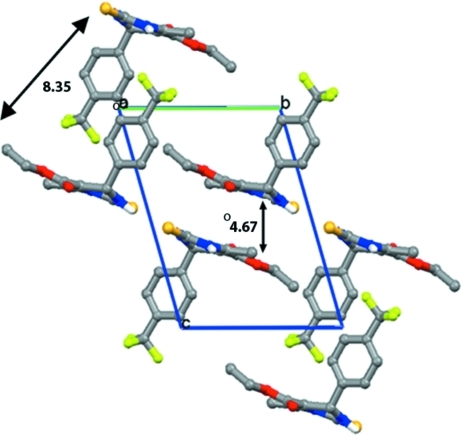
The molecular packing along the a axis with their polar and hydrophobic spacer distance (open circle represents centre of inversion).

## supplementary crystallographic information

### Comment

The synthesis of multifunctionlized dihydropyrimidone (DHPM) compounds was first reported by the Italian chemist Pietro Biginelli in 1893 (Kappe, 2000 and references therein). DHPMs have emerged as important target molecules because of their therapeutic and pharmacological properties such as anticarcinogenic (Kappe, 2000; Mayer *et al.*, 1999) and calcium channel modulators (Jauk *et al.*, 2000 and references therein). We have been working on this DHPMS system in view of the immense range of applications (Nayak *et al.*,2009; 2010). Here, we are reporting the single-crystal structure of the title compound.

The conformation of the molecule is controlled by an intra-molecular C—H···π interaction (2.655 (2) Å, Table 1) wherein the aryl hydrogen H14 is oriented towards the π electrons corresponding to the C2═C3 double bond (Fig. 1). The dihedral angles between the planes of 4-trifluoromethylphenyl and ester groups (O1/C5/O2/C6/C7) with the plane of the six-membered tetrahydropyrimidine ring are 81.8 (1)° and 16.0 (1)°, respectively. The crystal structure is mainly stabilized by hydrogen bonds of centrosymmetric N—H···S dimers and N—H···O molecular chains along the crystallographic *a* axis (Fig. 2). The molecules are arranged as polar and hyrophobic sheets with the spacer distances of 4.673 (2)Å and 8.359 (2) Å, respectively as briefly explained in literature (Fig. 3, Nayak *et al.*, 2009; 2010).

### Experimental

A mixture of ethylacetoacetate (0.1 mol), 4-trifluoromethylbenzaldehyde (0.1 mol) and thiourea (0.11 mol) was refluxed in 50 ml of ethanol for 2 h in the presence of concentrated hydrochloric acid as a catalyst. The reaction was monitored with thin layer chromatography and the reaction medium was quenched in ice cold water. The precipitate obtained was filtered, dried and crystallized from ethanol at room temperature to obtain the title compound.

### Refinement

All H atoms were located from difference Fourier map and refined freely with C–H and N–H distances in the range between 0.80Å and 1.0 Å.

### Crystal data

**Table:**

C_15_H_15_F_3_N_2_O_2_S	*Z* = 2
*M**_r_* = 344.36	*F*(000) = 356
Triclinic, *P*1	*D*_x_ = 1.506 Mg m^−^^3^
Hall symbol: -P 1	Melting point: 471(2) K
*a* = 7.2682 (3) Å	Mo *K*α radiation, λ = 0.71073 Å
*b* = 9.3205 (2) Å	Cell parameters from 467 reflections
*c* = 12.4779 (3) Å	θ = 1.0–27.9°
α = 74.199 (2)°	µ = 0.26 mm^−^^1^
β = 88.092 (3)°	*T* = 120 K
γ = 69.377 (3)°	Block, colorless
*V* = 759.39 (5) Å^3^	0.28 × 0.22 × 0.17 mm

### Data collection

**Table:**

Oxford Diffraction Xcalibur diffractometer with an Eos (Nova) detector	2982 independent reflections
Radiation source: Enhance (Mo) X-ray Source	2670 reflections with *I* > 2σ(*I*)
graphite	*R*_int_ = 0.034
Detector resolution: 16.0839 pixels mm^-1^	θ_max_ = 26.0°, θ_min_ = 2.4°
ω scans	*h* = −8→8
Absorption correction: multi-scan (*CrysAlis PRO*; Oxford Diffraction, 2009)	*k* = −11→11
*T*_min_ = 0.932, *T*_max_ = 0.958	*l* = −15→15
16986 measured reflections	

### Refinement

**Table:**

Refinement on *F*^2^	Primary atom site location: structure-invariant direct methods
Least-squares matrix: full	Secondary atom site location: difference Fourier map
*R*[*F*^2^ > 2σ(*F*^2^)] = 0.032	Hydrogen site location: inferred from neighbouring sites
*wR*(*F*^2^) = 0.085	All H-atom parameters refined
*S* = 1.05	*w* = 1/[σ^2^(*F*_o_^2^) + (0.0385*P*)^2^ + 0.4675*P*] where *P* = (*F*_o_^2^ + 2*F*_c_^2^)/3
2982 reflections	(Δ/σ)_max_ < 0.001
268 parameters	Δρ_max_ = 0.33 e Å^−^^3^
0 restraints	Δρ_min_ = −0.26 e Å^−^^3^

### Special details

**Table:**

Experimental. CrysAlisPro, Oxford Diffraction Ltd., Version 1.171.33.34d Empirical absorption correction using spherical harmonics, implemented in SCALE3 ABSPACK scaling algorithm.
Geometry. All s.u.'s (except the s.u. in the dihedral angle between two l.s. planes) are estimated using the full covariance matrix. The cell s.u.'s are taken into account individually in the estimation of s.u.'s in distances, angles and torsion angles; correlations between s.u.'s in cell parameters are only used when they are defined by crystal symmetry. An approximate (isotropic) treatment of cell s.u.'s is used for estimating s.u.'s involving l.s. planes.
Refinement. Refinement of *F*^2^ against ALL reflections. The weighted *R*-factor *wR* and goodness of fit *S* are based on *F*^2^, conventional *R*-factors *R* are based on *F*, with *F* set to zero for negative *F*^2^. The threshold expression of *F*^2^ > 2σ(*F*^2^) is used only for calculating *R*-factors(gt) *etc*. and is not relevant to the choice of reflections for refinement. *R*-factors based on *F*^2^ are statistically about twice as large as those based on *F*, and *R*- factors based on ALL data will be even larger.

### Fractional atomic coordinates and isotropic or equivalent isotropic displacement parameters (Å^2^)

**Table:**

	*x*	*y*	*z*	*U*_iso_*/*U*_eq_	
S1	0.30321 (6)	0.90692 (5)	0.45283 (3)	0.01866 (12)	
O2	−0.02059 (15)	0.41096 (13)	0.28311 (9)	0.0192 (2)	
N2	−0.01311 (19)	0.83739 (15)	0.42302 (10)	0.0152 (3)	
N1	0.2762 (2)	0.67834 (15)	0.37360 (10)	0.0152 (3)	
C3	−0.0065 (2)	0.61577 (17)	0.35436 (12)	0.0137 (3)	
O1	−0.28789 (15)	0.54256 (13)	0.35999 (9)	0.0202 (2)	
C9	−0.2190 (2)	0.89964 (17)	0.25202 (12)	0.0142 (3)	
C4	−0.1317 (2)	0.77821 (17)	0.36488 (12)	0.0139 (3)	
C5	−0.1193 (2)	0.52013 (17)	0.33520 (12)	0.0146 (3)	
C14	−0.1469 (2)	0.87476 (19)	0.15166 (13)	0.0179 (3)	
C1	0.1797 (2)	0.80426 (17)	0.41397 (12)	0.0144 (3)	
C2	0.1916 (2)	0.57285 (17)	0.35621 (12)	0.0139 (3)	
F3	−0.42949 (18)	1.20109 (14)	−0.14515 (8)	0.0412 (3)	
C6	−0.1217 (2)	0.31314 (19)	0.25754 (14)	0.0189 (3)	
C10	−0.3740 (2)	1.04101 (18)	0.25052 (13)	0.0170 (3)	
F2	−0.64945 (16)	1.33939 (13)	−0.05806 (9)	0.0389 (3)	
C11	−0.4529 (2)	1.15589 (19)	0.15063 (13)	0.0199 (3)	
C13	−0.2264 (2)	0.98893 (19)	0.05111 (13)	0.0202 (3)	
C8	0.3400 (2)	0.42067 (19)	0.34389 (15)	0.0196 (3)	
C12	−0.3786 (2)	1.12940 (19)	0.05093 (13)	0.0198 (3)	
C7	0.0075 (3)	0.2226 (2)	0.18351 (16)	0.0249 (4)	
F1	−0.3667 (2)	1.36503 (15)	−0.07403 (10)	0.0514 (4)	
C15	−0.4567 (3)	1.2573 (2)	−0.05606 (14)	0.0280 (4)	
H4	−0.246 (2)	0.7766 (19)	0.4098 (13)	0.010 (4)*	
H2N	−0.075 (3)	0.907 (2)	0.4538 (15)	0.016 (4)*	
H1N	0.393 (3)	0.652 (2)	0.3721 (16)	0.024 (5)*	
H10	−0.424 (3)	1.059 (2)	0.3189 (15)	0.016 (4)*	
H6B	−0.139 (3)	0.241 (2)	0.3277 (15)	0.016 (4)*	
H14	−0.045 (3)	0.779 (2)	0.1506 (15)	0.023 (5)*	
H13	−0.179 (3)	0.971 (2)	−0.0173 (16)	0.024 (5)*	
H6A	−0.251 (3)	0.384 (2)	0.2200 (16)	0.025 (5)*	
H11	−0.557 (3)	1.253 (2)	0.1503 (16)	0.026 (5)*	
H7A	0.021 (3)	0.296 (3)	0.1166 (18)	0.034 (5)*	
H7B	−0.054 (3)	0.152 (2)	0.1647 (17)	0.035 (5)*	
H7C	0.132 (3)	0.156 (3)	0.2222 (18)	0.037 (6)*	
H8B	0.292 (3)	0.332 (3)	0.3678 (17)	0.037 (6)*	
H8C	0.456 (3)	0.395 (3)	0.3874 (19)	0.042 (6)*	
H8A	0.367 (3)	0.431 (3)	0.265 (2)	0.049 (6)*	

### Atomic displacement parameters (Å^2^)

**Table:**

	*U*^11^	*U*^22^	*U*^33^	*U*^12^	*U*^13^	*U*^23^
S1	0.0165 (2)	0.0207 (2)	0.0237 (2)	−0.00844 (16)	0.00304 (15)	−0.01173 (16)
O2	0.0148 (5)	0.0196 (6)	0.0303 (6)	−0.0092 (5)	0.0051 (4)	−0.0143 (5)
N2	0.0131 (6)	0.0161 (6)	0.0180 (6)	−0.0042 (5)	0.0007 (5)	−0.0088 (5)
N1	0.0104 (7)	0.0178 (6)	0.0199 (6)	−0.0062 (5)	0.0035 (5)	−0.0080 (5)
C3	0.0144 (7)	0.0134 (7)	0.0133 (7)	−0.0049 (6)	0.0012 (5)	−0.0038 (5)
O1	0.0127 (6)	0.0223 (6)	0.0293 (6)	−0.0079 (5)	0.0048 (4)	−0.0114 (5)
C9	0.0124 (7)	0.0151 (7)	0.0175 (7)	−0.0074 (6)	0.0006 (6)	−0.0052 (6)
C4	0.0125 (7)	0.0162 (7)	0.0154 (7)	−0.0061 (6)	0.0027 (6)	−0.0072 (6)
C5	0.0141 (7)	0.0128 (7)	0.0147 (7)	−0.0032 (6)	−0.0009 (6)	−0.0023 (5)
C14	0.0161 (8)	0.0186 (8)	0.0205 (8)	−0.0053 (6)	0.0023 (6)	−0.0089 (6)
C1	0.0162 (7)	0.0156 (7)	0.0113 (7)	−0.0061 (6)	0.0016 (5)	−0.0030 (5)
C2	0.0156 (7)	0.0141 (7)	0.0124 (7)	−0.0061 (6)	0.0011 (5)	−0.0032 (5)
F3	0.0453 (7)	0.0503 (7)	0.0175 (5)	−0.0100 (6)	−0.0023 (5)	−0.0018 (5)
C6	0.0178 (8)	0.0185 (8)	0.0255 (8)	−0.0103 (7)	0.0016 (6)	−0.0089 (7)
C10	0.0144 (7)	0.0193 (8)	0.0193 (8)	−0.0063 (6)	0.0044 (6)	−0.0084 (6)
F2	0.0303 (6)	0.0363 (6)	0.0314 (6)	0.0008 (5)	−0.0071 (5)	0.0045 (5)
C11	0.0156 (8)	0.0165 (8)	0.0253 (8)	−0.0040 (6)	0.0003 (6)	−0.0043 (6)
C13	0.0219 (8)	0.0254 (8)	0.0166 (7)	−0.0109 (7)	0.0042 (6)	−0.0081 (6)
C8	0.0135 (8)	0.0174 (8)	0.0287 (9)	−0.0043 (6)	0.0014 (7)	−0.0092 (7)
C12	0.0176 (8)	0.0220 (8)	0.0201 (8)	−0.0092 (6)	−0.0020 (6)	−0.0031 (6)
C7	0.0233 (9)	0.0267 (9)	0.0333 (10)	−0.0137 (8)	0.0069 (7)	−0.0166 (8)
F1	0.0618 (9)	0.0444 (7)	0.0454 (7)	−0.0363 (7)	−0.0147 (6)	0.0168 (6)
C15	0.0277 (9)	0.0293 (9)	0.0243 (9)	−0.0120 (8)	−0.0022 (7)	−0.0005 (7)

### Geometric parameters (Å, °)

**Table:**

S1—C1	1.6863 (15)	F3—C15	1.334 (2)
O2—C5	1.3369 (18)	C6—C7	1.502 (2)
O2—C6	1.4557 (18)	C6—H6B	0.982 (18)
N2—C1	1.3323 (19)	C6—H6A	0.98 (2)
N2—C4	1.4725 (18)	C10—C11	1.385 (2)
N2—H2N	0.829 (19)	C10—H10	0.949 (18)
N1—C1	1.3572 (19)	F2—C15	1.336 (2)
N1—C2	1.3940 (19)	C11—C12	1.389 (2)
N1—H1N	0.80 (2)	C11—H11	0.95 (2)
C3—C2	1.351 (2)	C13—C12	1.384 (2)
C3—C5	1.472 (2)	C13—H13	0.946 (19)
C3—C4	1.506 (2)	C8—H8B	0.98 (2)
O1—C5	1.2128 (18)	C8—H8C	0.94 (2)
C9—C14	1.388 (2)	C8—H8A	0.99 (2)
C9—C10	1.396 (2)	C12—C15	1.494 (2)
C9—C4	1.534 (2)	C7—H7A	0.95 (2)
C4—H4	0.988 (17)	C7—H7B	1.00 (2)
C14—C13	1.389 (2)	C7—H7C	0.95 (2)
C14—H14	0.941 (19)	F1—C15	1.348 (2)
C2—C8	1.493 (2)		
			
C5—O2—C6	117.51 (12)	O2—C6—H6A	108.1 (11)
C1—N2—C4	123.64 (13)	C7—C6—H6A	112.1 (11)
C1—N2—H2N	119.0 (13)	H6B—C6—H6A	109.9 (15)
C4—N2—H2N	116.5 (13)	C11—C10—C9	120.59 (14)
C1—N1—C2	123.78 (13)	C11—C10—H10	120.0 (10)
C1—N1—H1N	118.1 (14)	C9—C10—H10	119.4 (10)
C2—N1—H1N	116.8 (14)	C10—C11—C12	119.70 (15)
C2—C3—C5	125.59 (13)	C10—C11—H11	120.2 (12)
C2—C3—C4	119.99 (13)	C12—C11—H11	120.1 (11)
C5—C3—C4	114.30 (12)	C12—C13—C14	119.71 (15)
C14—C9—C10	118.99 (14)	C12—C13—H13	119.8 (11)
C14—C9—C4	122.22 (13)	C14—C13—H13	120.5 (11)
C10—C9—C4	118.79 (13)	C2—C8—H8B	111.7 (12)
N2—C4—C3	109.18 (12)	C2—C8—H8C	109.6 (13)
N2—C4—C9	109.86 (11)	H8B—C8—H8C	108.3 (18)
C3—C4—C9	113.06 (12)	C2—C8—H8A	110.1 (14)
N2—C4—H4	106.2 (9)	H8B—C8—H8A	106.3 (18)
C3—C4—H4	112.9 (9)	H8C—C8—H8A	110.7 (19)
C9—C4—H4	105.4 (9)	C13—C12—C11	120.31 (15)
O1—C5—O2	123.28 (13)	C13—C12—C15	120.14 (15)
O1—C5—C3	123.34 (13)	C11—C12—C15	119.47 (15)
O2—C5—C3	113.31 (12)	C6—C7—H7A	109.3 (12)
C9—C14—C13	120.70 (15)	C6—C7—H7B	109.3 (12)
C9—C14—H14	120.4 (11)	H7A—C7—H7B	109.4 (17)
C13—C14—H14	118.9 (11)	C6—C7—H7C	109.8 (13)
N2—C1—N1	116.24 (13)	H7A—C7—H7C	111.7 (18)
N2—C1—S1	123.24 (11)	H7B—C7—H7C	107.3 (17)
N1—C1—S1	120.50 (11)	F3—C15—F2	106.80 (14)
C3—C2—N1	118.64 (13)	F3—C15—F1	106.00 (15)
C3—C2—C8	128.19 (14)	F2—C15—F1	106.29 (15)
N1—C2—C8	113.16 (13)	F3—C15—C12	112.93 (15)
O2—C6—C7	106.10 (12)	F2—C15—C12	112.89 (14)
O2—C6—H6B	108.8 (10)	F1—C15—C12	111.43 (14)
C7—C6—H6B	111.6 (10)		
			
C1—N2—C4—C3	32.85 (18)	C5—C3—C2—N1	178.91 (13)
C1—N2—C4—C9	−91.67 (16)	C4—C3—C2—N1	3.2 (2)
C2—C3—C4—N2	−24.38 (18)	C5—C3—C2—C8	−2.4 (2)
C5—C3—C4—N2	159.44 (12)	C4—C3—C2—C8	−178.11 (14)
C2—C3—C4—C9	98.24 (16)	C1—N1—C2—C3	15.8 (2)
C5—C3—C4—C9	−77.94 (15)	C1—N1—C2—C8	−163.11 (14)
C14—C9—C4—N2	105.73 (15)	C5—O2—C6—C7	170.73 (13)
C10—C9—C4—N2	−73.24 (16)	C14—C9—C10—C11	−1.0 (2)
C14—C9—C4—C3	−16.51 (19)	C4—C9—C10—C11	177.99 (13)
C10—C9—C4—C3	164.51 (13)	C9—C10—C11—C12	0.8 (2)
C6—O2—C5—O1	−1.3 (2)	C9—C14—C13—C12	0.3 (2)
C6—O2—C5—C3	−178.35 (12)	C14—C13—C12—C11	−0.5 (2)
C2—C3—C5—O1	161.70 (15)	C14—C13—C12—C15	176.18 (15)
C4—C3—C5—O1	−22.4 (2)	C10—C11—C12—C13	0.0 (2)
C2—C3—C5—O2	−21.2 (2)	C10—C11—C12—C15	−176.74 (15)
C4—C3—C5—O2	154.72 (12)	C13—C12—C15—F3	25.8 (2)
C10—C9—C14—C13	0.5 (2)	C11—C12—C15—F3	−157.42 (15)
C4—C9—C14—C13	−178.50 (13)	C13—C12—C15—F2	147.12 (15)
C4—N2—C1—N1	−17.6 (2)	C11—C12—C15—F2	−36.1 (2)
C4—N2—C1—S1	163.99 (11)	C13—C12—C15—F1	−93.35 (19)
C2—N1—C1—N2	−8.8 (2)	C11—C12—C15—F1	83.40 (19)
C2—N1—C1—S1	169.61 (11)		

### Hydrogen-bond geometry (Å, °)

**Table:**

*D*—H···*A*	*D*—H	H···*A*	*D*···*A*	*D*—H···*A*
N1—H1N···O1^i^	0.80 (2)	2.20 (2)	2.9868 (19)	169.8 (18)
N2—H2N···S1^ii^	0.830 (19)	2.462 (19)	3.2830 (14)	170 (2)
C14—H14···Cg1	0.94 (2)	2.655 (2)	3.129 (2)	112

Symmetry codes: (i) *x*+1, *y*, *z*; (ii) −*x*, −*y*+2, −*z*+1.
